# Gaming and social media use among adolescents in the midst of the COVID-19 pandemic

**DOI:** 10.1177/14550725221074997

**Published:** 2022-02-04

**Authors:** Anders Nilsson, Ingvar Rosendahl, Nitya Jayaram-Lindström

**Affiliations:** Karolinska Institutet & Stockholm Health Care Services, Stockholm County Council, Sweden; Karolinska Institutet & Stockholm Health Care Services, Stockholm County Council, Sweden; Karolinska Institutet & Stockholm Health Care Services, Stockholm County Council, Sweden

**Keywords:** adolescents, COVID-19, gaming, social media

## Abstract

**Background and aims:** The COVID-19 pandemic has dramatically changed life circumstances for adolescents worldwide. With schools being closed and regular activities being cancelled, gaming and social media use are activities that might gain in importance. There is a risk that these online behaviours have negative effects on other important activities, such as being physically active, sleeping, and studying, as well as general well-being. The aim of this study was to investigate the effects of the COVID-19 pandemic on gaming and social media use, and its effects on the well-being of adolescents. **Methods:** A total of 1232 adolescents (82.5% female) participated in an anonymous web survey containing questions on gaming, social media use, and perceived negative consequences of gaming and social media use during the COVID-19 pandemic. The results were analysed with a quasi-Poisson regression model. **Results:** The results indicated an increase in gaming and social media use, which was associated with negative consequences and perceived well-being. A majority of adolescents reported that they used social media more than they felt comfortable with. There were large differences between boys and girls, with girls being more negatively affected across measures. **Discussion:** The increase in gaming and social media use during the COVID-19 pandemic might have negative effects on the well-being of adolescents, and on other activities that are important to health. Our interpretation of the findings is that gaming and social media use might partly function as coping mechanisms to deal with stress and/or boredom resulting from COVID-19 restrictions. There is a risk that these coping strategies become maladaptive over time. **Conclusions:** The restrictions from the COVID-19 pandemic have resulted in an increase in the amount of time adolescents spend gaming and on social media, which might have negative effects on their well-being. This study highlights the urgent need to consider adaptive and healthy coping strategies for adolescents given the pandemic may mean that daily living could continue to be altered in the near future.

During the initial phases of the COVID-19 pandemic, the behaviour patterns of billions of people around the world changed dramatically. Health authorities urged individuals to stay at home to help prevent the spread of the virus, and as a result a wide range of activities were either prohibited, severely limited, or markedly altered. Sweden gained some attention for its government's decision to impose relatively mild measures, compared to many other similar countries. Nevertheless, during the initial phases of the COVID-19 pandemic, from the end of March 2020 and onwards, the Swedish government imposed travel restrictions within and outside the country, limited the number of people allowed in large gatherings, advised all universities and high schools to pursue home-based online schooling, as well as urging everyone who could to work from home and avoid unnecessary social contact. While high school students were instructed to study from home, schools for younger children were largely kept open. However, all after-school and extracurricular activities were to a large extend cancelled. Data from telephone companies tracking mobile phone signals indicated that the movements of the general public decreased by approximately 20%, and up to 35% in the bigger cities, during the months of March and April 2020 (Telia, 2020).

A number of studies have investigated the adverse effects on mental health due to the COVID-19 pandemic (Chen et al., 2021; Kwong et al., 2021; O’Connor et al., 2021; Panchal et al., 2020; Sher, 2020). While the long-term effects of the pandemic on mental health are continuously analysed, studies on the effects of the initial phases of the COVID-19 pandemic reveal that it poses a serious threat to general public mental health. Isolation, anxiety, fear of contagion, depression and insomnia are among the mental health problems associated with the COVID-19 pandemic (Hao et al., 2020; Huang & Zhao, 2020; Qiu et al., 2020).

With many other social activities coming to a halt, activities available at home, such as gaming and using social media, saw an upswing during the initial stages of the pandemic. For example, online gaming in the US increased 75% during initial stay-at-home directives (Hollywood Reporter, 2020), and Fortnite-related gaming was up 70% in Italy (Bloomberg News, 2020). Despite not implementing outright lockdowns, gaming increased by 23% in Scandinavia during April 2020 (SVT Nyheter, 2020). Online gaming was already one of the most common spare time activities among Swedish youth aged 13–16 years, and the number of daily social media users reach 84% in the same age group (Statens Medieråd, 2019). On average, 14–17-year-olds spend ten hours a week playing computer games, and another three hours watching others play (Sovré & Robertson, 2017). Engaging in gaming and social media are in many ways examples of highly suitable activities in a time when people are urged, or forced, to stay at home.

There is, however, a possible downside to an increase in the time spent gaming or using social media. Several studies (Andreassen et al., 2016; Boers et al., 2019; Brunborg et al., 2013; Twenge et al., 2018) have found an association between time spent gaming or on social media, and psychological distress such as depression and anxiety, sleep disturbances, poor hygiene, poor diet, and lower academic achievement (Brunborg et al., 2014; Brunborg et al., 2013; Carey et al., 2021). Other studies, however, point to no or small such effects (Berryman et al., 2018; Orben et al., 2019; Orben & Przybylski, 2019), or to the possibility of an inverted U-curve where screen use is beneficial for health and psychosocial functioning up to a certain point, where it starts to have mostly negative effects (Przybylski et al., 2020).

While gaming and social media use per se differ in many aspects, the World Health Organization label them both as examples of *sedentary screen use* (World Health Organization, 2019)*.* Furthermore, certain platforms, such as Twitch and Discord, blur the lines between social media and gaming since they offer both gaming and social interaction. Recent years have seen an increased scientific interest in the relationship between different types of screen activities, such as gaming or social media use, and various health outcomes, as well as possible consequences for school results, physical activity or relationships to others. The most substantial outcome of these studies is perhaps the creation of a new diagnosis in International Classification of Diseases 11^th^ Revision (ICD-11): *gaming disorder* (Reed et al., 2019).

However, the creation of this novel disorder has been hotly debated (Aarseth et al., 2017; Király & Demetrovics, 2017; Rumpf et al., 2018; Van Rooij et al., 2018), and the very assumption that screen activities are causing ill-health has also been disputed (Orben & Przybylski, 2019). The main objection has been that an association does not imply causation, and that there is a risk that such a diagnosis would pathologize and stigmatise otherwise harmless activities. Several studies have also identified possible benefits of gaming, such as enriching people's lives (Granic et al., 2014) and reducing loneliness (Carras et al., 2017). The critique against the gaming disorder diagnoses also stems from what is seen as a lack of well-designed studies in the field (Aarseth et al., 2017).

The lockdowns during the COVID-19 pandemic offer a rare opportunity to examine instantaneous, and nationwide, behaviour changes. Some preliminary reports have indeed indicated a possible increase in problematic gaming as a result of the COVID-19 pandemic (Balhara et al., 2020; Fazeli et al., 2020) as well as problematic screen time use (Fernandes et al., 2020).

Given the association between gaming, social media use and psychological distress, it is relevant to further investigate this link at a time when such activities are likely to gain in importance. This study investigates changes in gaming and social media use among a sample of adolescents, and its possible link to psychological distress, within the Swedish context.

## Research questions

- Has the COVID-19 pandemic led to an increase in gaming and social media use?
- Is an assumed change in gaming habits and social media use after the start of the COVID-19 pandemic different between the sexes or/and different ages?
- Is there an association between time spent on gaming and/or social media and psychological distress and/or other negative consequences?
- Are there any differences in psychological distress and/or other negative consequences from gaming and/or social media use between the sexes or/and different ages?

## Methods

The present study is a cross-sectional, nationwide study based on an online survey on consequences of the COVID-19 pandemic, and its relation to activities such as gaming and social media use distributed to adolescents in Sweden.

### Participants

The participants (*N* = 1232) were adolescents aged 13–18 years. Adolescents aged 13–15 years usually attend junior high school, which is compulsory, and adolescents aged 16–18 years usually attend high school, which is not compulsory but still initiated by 99% of students in Sweden (Statistiska Centralbyrån, 2017).

### Procedure

The participants were either recruited through schools across the country or through study advertisements on Facebook. The participants had to agree that their answers could be collected and analysed within the framework of the present study, but they were also informed that they could end participation at any time during the survey. The survey was completely anonymous, and no personal information was collected (including email addresses or names). The participants were not offered any reimbursement for their participation. The data were collected from 5 June to 10 August 2020, corresponding to the first wave of the pandemic.

### Measures

The survey consisted of 40 questions on demographics such as age, gender, and living conditions, consequences of the COVID-19 pandemic (e.g., being ill from COVID-19, school closing or spare time activities being cancelled), gaming, social media use, and perceived negative consequences of gaming and social media use. See Table 1 for a full list of questions on regular habits and patterns of gaming and use of social media and possible negative consequences from gaming or social media in relation to the pandemic.

**Table 1.** Questions on negative consequences of gaming and social media use along with questions on gaming and social media activity.

Question	Possible answers
Have you been gaming/using social media more than you would have liked (i.e., felt was good for you)?	1. Yes, more than I would have wanted. 2. Yes, less than I wanted. 3. Neither more nor less than I wanted.
To what degree has your well-being been negatively affected (e.g., being worried, depressed or experienced anxiety) because of your gaming/social media use during the corona/COVID-19 pandemic?	1–5 where 1 indicates *Not at all* and 5 indicates *Completely.*
Indicate if your gaming/social media use had a negative effect on any of the following BEFORE the corona/COVID-19 pandemic:	- Your studies - Your planned activities - How much you are physically active - How you’re feeling - Your sleep - Your economy - Your relationships with your finances - Your other interests - Your relationship with your family (e.g., conflicts with your parents, spending less time with your family)
Indicate if your gaming/social media use had a negative effect on any of the following since the corona/COVID-19 pandemic started:	- Your studies - Your planned activities - How much you are physically active - How you’re feeling - Your sleep - Your economy - Your relationships with your finances - Your other interests - Your relationship with your family (e.g., conflicts with your parents, spending less time with your family)
Do you usually play computer or video games?	- Never - Sometimes - Often
Approximately how many hours per day have you played computer or video games in the last two weeks?	- Not at all - 0–2 hours per day - 2–4 hours per day - 4–6 hours per day - 6–8 hours per day - 8+ hours per day
Is it a difference from in normal cases (before the COVID-19 pandemic)?	- Yes, less - Yes, more - No
Have you played more during the day than before the COVID-19 pandemic?	- No - Yes
Have you played more in the evening than before the COVID-19 pandemic?	- No - Yes
Have you played more at night than before the COVID-19 pandemic?	- No - Yes
Have you played more or less than you would have liked?	- Yes, more - Yes, less - No
How much time per day have you spent on social media (enter in integers)?	- Not a categorical scale
Is it a difference from in normal cases (before the COVID-19 pandemic)?	- Yes, less - Yes, more - About the same
Have you spent more or less time on social media than you would have liked?	- Yes, more - Yes, less - No

### Statistical analysis

In the analytical part of the study, the outcome was degree of negative consequences. The question was answered by the adolescents on an ordinal scale ranging from 1 (not at all) to 5 (completely), which makes it suitable to analyse with a Poisson regression model after recoding the scale to range from 0 to 4 points. However, overdispersion occurs in Poisson regression when the observed variance of the outcome is larger than would be predicted by the Poisson distribution. If overdispersion is present and not accounted for in the analysis, the resulting standard errors are too small. The presence of overdispersion was tested by calculating the ratio of the residual deviance to the residual degrees of freedom and if this ratio was substantially larger than 1, overdispersion was on good ground assumed and a quasi-Poisson approach was used instead (Kabacoff, 2010). For all descriptive statistics and all analyses, the free statistical software R (Core Team R, 2013) was used.

### Ethics

The study procedures were carried out in accordance with the Declaration of Helsinki (World Medical Association, 2013). The study was reviewed by the Swedish Ethical Review Authority (file number 2020-02556), and deemed not to require ethics approval since the data in the study cannot be directly or indirectly linked to an individual.

## Results

Table 2 shows the background characteristics for the adolescents, where the high difference in number of girls and boys who completed the survey was most noticeable.

**Table 2.** Background characteristics among the 1232 adolescents.

Characteristics	*N*	%
Legal gender		
Girls	1016	82.5
Boys	201	16.3
Do not want to answer	15	1.2
Age (in years)		
13	24	1.9
14	150	12.2
15	153	12.4
16	246	20.0
17	359	29.1
18	300	24.4
Employment		
High school (Grades 7–9)	386	31.3
Senior High School (Grades 10–12)	819	66.5
Working	14	1.1
Neither goes to school or works	13	1.1
Urban/rural area		
City	287	23.3
Town	240	19.5
Small town	237	19.2
Smaller urban area	218	17.7
Village/countryside	250	20.3
Form of housing		
Apartment	277	22.5
Row house	120	9.8
Detached house	795	64.5
Other accommodation	40	3.2
Lives with		
Full time with mom and dad	731	59.3
Full time with mom	200	16.2
Full time with dad	42	3.4
Alternately with mom and dad	215	17.5
Other people	27	2.2
By myself	17	1.4
Access to own room		
Full time	1170	95.0
Part time	41	3.3
Not at all	21	1.7
Access to own computer		
Full time	915	74.3
Part time	104	8.4
Not at all	213	17.3
Access to own mobile phone		
Full time	1229	99.8
Part time	0	0.0
Not at all	3	0.2

Table 3 presents frequency of gaming and social media use separated by sex, where the gaming part of the survey had a filter question about whether the adolescent was gaming at all or not. There was a substantial difference between the sexes regarding gaming participation. More than 60% of the girls were not gaming at all, the same figure among boys was 13%. However, among those who were gaming, the sex differences in increases in gaming during the COVID-19 pandemic were less striking. The proportion who had been gaming more than they felt comfortable with was almost identical between the sexes.

**Table 3.** Gaming and social media use and changes among the 1217 adolescents during the pandemic (numbers in parentheses are the proportions).

Gaming	Boys	Girls
Existing	*n* = 201	*n* = 1016
Never	26 (12.9)	633 (62.3)
Sometimes	59 (29.4)	316 (31.1)
Often	116 (57.7)	67 (6.6)
Number of hours spent gaming per day last two weeks	*n* = 175	*n* = 383
0	7 (4.0)	70 (18.3)
1–2	53 (30.3)	167 (43.6)
3–4	48 (27.4)	76 (19.8)
5–6	32 (18.3)	35 ((9.1)
7–8	18 (10.3)	19 (5.0)
8+	17 (9.7)	16 (4.2)
Difference in time gaming from before the pandemic		
Gaming less	11 (6.3)	22 (5.7)
Gaming more	56 (32.0)	105 (27.4)
No difference	108 (61.7)	256 (66.9)
Difference gaming times compared to before the pandemic^a^	*n* = 56	*n* = 105
More during daytime	34 (60.7)	57 (54.3)
More in the evening	17 (30.4)	31 (29.5)
More during nighttime	15 (26.8)	24 (22.9)
Opinions own gaming	*n* = 175	*n* = 383
Been gaming more than felt ok	39 (22.3)	83 (21.7)
Been gaming less than felt ok	14 (8.0)	16 (4.2)
Been gaming neither too much nor too little	122 (69.7)	284 (74.1)
**Social media use**	**Boys**	**Girls**
Frequency	*n* = 192	*n* = 987
Median number of hours spent on social media per day	3	5
Mean number of hours spent on social media per day	3.9	5.6
*SD*	2.71	3.17
Difference from the pandemic	*n* = 201	*n* = 1016
Decreased time spent on social media	9 (4.5)	41 (4.0)
Increased time spent on social media	69 (34.3)	568 (55.9)
No difference	123 (61.2)	407 (40.1)
Opinions own social media use		
Used social media more than felt ok	73 (36.3)	629 (61.9)
Used social media less than felt ok	4 (2.0)	35 (3.4)
Used social media neither too much nor too little	124 (61.7)	352 (34.6)

^a^ Response alternatives are not multiple exclusively.

No filter question was present for the part about social media use, assuming that all adolescents use social media, since most of the participants were recruited through social media. While this assumption turned out to be true (100% of participants reported using at least one social media platform), the use of social media and the consequences of the COVID-19 pandemic on its use were much more present among the girls. Nearly 62% of the girls felt that they had used social media more than they felt comfortable with, while the corresponding percentage among boys was 36. The most common social media platforms used were Snapchat, used by 97% of participants, Instagram (96%), YouTube (92%), Facebook (82%) and TikTok (80%).

Questions on negative consequences from gaming and use of social media were asked in the survey, and Table 4 presents the presence of different types of negative consequences. A negative impact on sleep was the most common negative consequences from both gaming and use of social media, whereas there was a large difference in negative impact on studies where the use of social media had twice as high a negative impact on studies compared to gaming. However, since the impact on studies would most likely have been very different between high school (grades 7–9) students and senior high school (grades 10–12) students we also separated the two groups in Table 4, and there was a huge difference in the comparison of the presence of negative consequences on studies between pre- and post-pandemic. The high school students reported a lower presence of negative consequences for studies from both gaming and social media use for the period during pandemic compared to period before the pandemic, whereas the senior high school students instead reported the completely opposite change between the two periods with a higher presence of negative consequences for studies from both gaming and social media use during the pandemic period compared to the period before the pandemic.

**Table 4.** Presence (%) of different types of negative consequences from gaming and use of social media.

	Gaming (*n* = 570^a^)		Social media (*n* = 1232^b^)	
Negative consequences for:	Before	During	Before	During
Studies	21.9	23.5	42.6	45.6
Other activities	6.0	10.9	10.4	17.5
Physical activity	33.7	36.7	38.0	42.1
Well-being	11.1	15.6	36.9	40.5
Sleep	34.4	34.9	55.3	56.9
Economy	4.1	4.0	3.5	5.7
Relationships, finances	8.8	10.9	14.2	18.9
Relationships, family	15.6	14.2	20.1	17.0
Other interests	7.5	10.4	11.2	12.1
Studies (Grades 7–9)	27.6	19.8	38.1	28.8
Studies (Grades 10–12)	18.8	26.6	44.8	53.7

^a^ Observations for gaming, Grades 7–9 *n* = 222 and Grades 10–12 *n* = 335.

^b^ Observations for social media, Grades 7–9 *n* = 386 and Grades 10–12 *n* = 819.

As the outcome in our analyses, we used the question of the extent to which gaming and use of social media had affected the adolescents’ mood/well-being. We initially tried a Poisson regression model for the data but found that the outcome was highly overdispersed, and instead we therefore used a quasi-Poisson model for our analyses. Besides sex and age, three measures of gaming and use of social media with increasing sharpness were used as independent variables: frequency, increase in use during the pandemic, and whether the use was on a higher level than the adolescent felt comfortable with. The sharper the question was asked on grade of gambling or/and social media use, the higher the relative risk (RR) of negative consequences from gambling and social media use compared to those who had not increased their gambling or/and social media use during the COVID-19 pandemic. The RR for negative consequences from gambling and social media use between ages was negligible, although statistically significantly higher with increasing age, while there was a noticeably lower RR for boys compared to girls. The RR (risk ratio, or relative risk), compares the risk of some event (positive or negative) for one group often called “exposed” to a “non-exposed” group by dividing the incidence proportion or attack rate among the exposed with the risk in the non-exposed group. If the RR value is above 1, the risk is higher for the exposed and if the RR value is below 1 the risk is lower for the exposed and finally, if the RR is exactly 1 then the risk is the same in the two groups. However, there is a difference in interpretation of the RR estimate, dependent on whether the exposure is dichotomous or on a continuous scale. For instance, in Table 5 the exposure *Been gaming more than felt ok/Used social media more than felt ok* and being a boy are dichotomous exposures and the RR values then imply that it is more than three times higher risk (RR = 3.25, *p* < 0.001) for those who have played more than felt ok to have been affected negatively compared to those who have not been gaming more than felt ok. The consequences of having used social media more than felt ok have a value of RR = 1.88 (*p* < 0.001), which means there is an 88% higher risk of being affected negatively. Boys have a lower risk for the outcome by 1–0.69 = 0.31 * 100 = 31 per cent in the model for the variable *opinions own gaming* and 1–0.54 = 0.46 * 100 = 46 per cent in the model for the variable *opinions own social media use*. However, age is on a continuous scale and the interpretation of the RR value is the increase in RR for one standard deviation increase in age and is 1.09 (*p* = 0.041) for the variable *opinions own gaming* and 1.06 (*p* = 0.001) for the variable *opinions own social media use*.

**Table 5.** Quasi-Poisson regression analyses of potential risk from extensive gaming (*n* = 558) and social media use (*n* = 1217) on degree of negative consequences of gaming and social media use, adjusted for sex and age.

Gaming	Estimate	*SD*	*t*	*P*	RR
Frequency of gaming					
Intercept	–0.81	0.150	–5.38	< 0.001	0.45
Hours per day gaming	0.24	0.043	5.61	< 0.001	1.27
Boys	–0.56	0.154	–3.63	< 0.001	0.57
Age (centred)	0.09	0.043	2.03	0.043	1.09
Increased gaming during the pandemic					
Intercept	–0.53	0.100	–5.32	< 0.001	0.59
Have been gaming more	0.96	0.126	7.57	< 0.001	2.60
Boys	–0.43	0.149	–2.88	0.004	0.65
Age (centred)	0.06	0.043	1.32	0.188	1.06
Opinions own gaming					
Intercept	–0.55	0.097	–5.65	< 0.001	0.58
Been gaming more than felt ok	1.18	0.126	9.36	< 0.001	3.25
Boys	–0.37	0.150	–2.43	0.016	0.69
Age (centred)	0.09	0.043	2.04	0.041	1.09
**Social media**	**Estimate**	***SD***	***t***	***P***	**RR**
Frequency of social media use					
Intercept	0.26	0.053	4.94	< 0.001	1.30
Hours per day using social media	0.04	0.008	4.94	< 0.001	1.04
Boys	–0.69	0.094	–7.37	< 0.001	0.50
Age (centred)	0.09	0.020	4.43	< 0.001	1.09
Increased use of social media during the pandemic					
Intercept	0.15	0.046	3.19	0.001	1.16
Used social media more	0.50	0.053	9.45	< 0.001	1.65
Boys	–0.67	0.091	–7.41	< 0.001	0.51
Age (centred)	0.06	0.019	3.05	0.002	1.06
Opinions own social media use					
Intercept	0.02	0.051	0.42	0.672	1.02
Used social media more than felt ok	0.63	0.056	11.22	< 0.001	1.88
Boys	–0.62	0.091	–6.85	< 0.001	0.54
Age (centred)	0.06	0.019	3.27	0.001	1.06

## Discussion

The results of this nationwide study in Sweden show that the first wave of the COVID-19 pandemic has led to an increase in gaming and social media use among adolescents. The results also point to an association between an increase in these digital behaviours and negative consequences. Furthermore, the adolescents in the study report that they had been gaming and using social media more than they would have wanted, and that their social media use had to some extent affected their well-being negatively.

In this study evaluating the effects of the first wave of the COVID-19 pandemic on adolescents’ use of gaming and social media, a majority of adolescents used social media more than they wanted to, and a little more than a fifth of adolescents who game felt they had been gaming more than they wanted to. This greatly increased the risk of experiencing negative consequences from social media and/or gaming. Gaming and social media were found to negatively affect behaviours that are intrinsic in promoting mental health resilience, such as getting enough sleep and being physically active. Several other behaviours that are important to mental health in this age group were restricted during the first wave of the pandemic, such as meeting friends and participating in organised sports. This makes it concerning that gaming and social media use might to some extent further limit what healthy behaviours the adolescents would otherwise partake in.

The current study highlights a possible link between gaming, social media use and mental ill-health in this age group, but the results need to be interpreted with caution. The COVID-19 pandemic has, for many of the included adolescents, affected life in multiple ways. It is likely that some of the negative consequences associated with gaming or social media are in reality related to consequences of the pandemic, such as school closings, restriction in extracurricular activities, health concerns or economic stress for the family. Although the current study did not pose questions on the time parents spent with the adolescents, the increase in gaming and social media can also be related to dramatic changes within the home environment as a result of this pandemic, such as parents working from home and juggling other responsibilities.

Some specific results are worth further consideration. Across measurements, being male seemed to be protective of experiencing negative consequences related to gaming or social media use. This could be due to a selection bias, since the vast majority of adolescents who volunteered to take part in the study were female. It could reflect a tendency among young males not to reveal their vulnerability or to deny any problems connected to gaming, in line with previous research findings on male problem denial and reluctance to seek treatment for psychological problems (Galdas et al., 2005). However, some previous studies have suggested that adolescent girls are more negatively affected by social media than boys (McCrae et al., 2017).

A surprising finding was the weak association between time spent on social media and negative consequences, especially since an *increase* in social media use was associated with more negative consequences. It is possible that a high overall social media consumption also indicates a high level of social interaction with friends (Nesi et al., 2018), which could be beneficial in a time when there are few other social arenas. An increase in the use of social media might, on the other hand, reflect coping strategies in the face of changes stemming from the COVID-19 pandemic. Previous studies have found mixed results regarding time spent on social media and psychological well-being, either finding no meaningful effects (Orben et al., 2019; Orben & Przybylski, 2019), or quite large effects (Boers et al., 2019). It has also been suggested that “screen time”, a more loosely defined concept including various forms of activities using screens, follow a U-shaped curve where time spent on screen activities is associated with increased psychosocial functioning up to a certain point, before being associated with more negative consequences (Przybylski et al., 2020). Future studies will need to delve deeper to examine the differences between different types of social media, time spent, its relevance and gender differences.

There are some differences in the results between gaming and social media worth highlighting. The WHO term *sedentary screen time* assumes that social media and gaming are similar in terms of the negative effects they can cause. However, a higher share of adolescents in this study experienced negative consequences from social media than from gaming, especially regarding studies, well-being and sleep. This is worth pointing out, since gaming is often associated with these types of harms (Brunborg et al., 2013; Gnambs et al., 2020; Hale & Guan, 2015; Vadlin et al., 2016). One possible explanation is that gaming is more of a well-defined activity with clear boundaries as to when and where it is pursued, while social media use tends to be fluid, continuous and varies in what it constitutes (Nesi et al., 2018). In other words, it is more probable to be involuntarily interrupted by social media messages and notifications while sleeping and studying than by gaming elements.

Various screen time activities, such as gaming, gambling, social media use and online shopping have been suggested to function as coping strategies for adolescents in the face of stress related to the COVID-19 pandemic (Fernandes et al., 2020). While not inherently harmful, these coping strategies might become maladaptive over time (Fernandes et al., 2020). This also highlights some of the potential health risks associated with measures to combat the COVID-19 pandemic, such as curfews and home schooling.

## Limitations

The intention to capture a phenomenon within a limited timeframe had an effect on the choice of study design, and what conclusions can be drawn from the results. The cross-sectional design offers a snapshot view of the situation during the pandemic, but is unable to draw conclusions on long-term effects. The study is based on a self-recruited and anonymous group, which had an uneven gender representation. Since the sample is non-representative, there are limits to what generalisations can be drawn to the general population. The survey was anonymous, and there is thus a risk that some participants did not accurately state background information regarding age, sex or place of residence.

Some of the respondents filled out the questionnaire in the summer months when the schools were closed, which could have affected their answers, in particular regarding implications for their studies. However, the participants were instructed to report any consequences they had during the pandemic and the changes brought by it, and it can thus be argued that these responses are reflective of the time period that constitutes the first wave of the pandemic.

With the limited timeframe in mind, no validated diagnostic measures were used. Using validated measurements would have increased what conclusions can be drawn from the results, but would also have required a longer processing time of the ethical application (see also the Ethics section).

The question regarding time spent on social media could for some have been understood as a question regarding all screen time.

## Conclusions

Gaming and social media use are a mainstream form of recreation across age groups, and specifically among adolescents, and the risk of problematising the behaviour is high. The COVID-19 pandemic has led to rapid changes and restrictions in the lives of adolescents, which also provided an opportunity to study their engagement in gaming and social media during these circumstances. Our results highlighted that within a large community sample, there is a subgroup of adolescents who tended to spend excessive time gaming and consequently also experienced a decline in their mental health and well-being. Excessive time spent gaming and using social media may be a coping mechanism for adolescents to deal with the stress and/or boredom resulting from the restrictions. It is therefore prudent that the parents recognise the warning signs and monitor their children to help them identify adaptative coping strategies to deal with the challenges arising due to the COVID-19 pandemic.
